# B cell receptor repertoire abnormalities in autoimmune disease

**DOI:** 10.3389/fimmu.2024.1326823

**Published:** 2024-01-31

**Authors:** Hayato Yuuki, Takahiro Itamiya, Yasuo Nagafuchi, Mineto Ota, Keishi Fujio

**Affiliations:** ^1^ Department of Allergy and Rheumatology, Graduate School of Medicine, The University of Tokyo, Tokyo, Japan; ^2^ Department of Functional Genomics and Immunological Diseases, Graduate School of Medicine, The University of Tokyo, Tokyo, Japan

**Keywords:** BCR repertoire, repertoire analysis, autoimmune disease, unswitched memory B cell, B cells

## Abstract

B cells play a crucial role in the immune response and contribute to various autoimmune diseases. Recent studies have revealed abnormalities in the B cell receptor (BCR) repertoire of patients with autoimmune diseases, with distinct features observed among different diseases and B cell subsets. Classically, BCR repertoire was used as an identifier of distinct antigen-specific clonotypes, but the recent advancement of analyzing large-scale repertoire has enabled us to use it as a tool for characterizing cellular biology. In this review, we provide an overview of the BCR repertoire in autoimmune diseases incorporating insights from our latest research findings. In systemic lupus erythematosus (SLE), we observed a significant skew in the usage of VDJ genes, particularly in CD27^+^IgD^+^ unswitched memory B cells and plasmablasts. Notably, autoreactive clones within unswitched memory B cells were found to be increased and strongly associated with disease activity, underscoring the clinical significance of this subset. Similarly, various abnormalities in the BCR repertoire have been reported in other autoimmune diseases such as rheumatoid arthritis. Thus, BCR repertoire analysis holds potential for enhancing our understanding of the underlying mechanisms involved in autoimmune diseases. Moreover, it has the potential to predict treatment effects and identify therapeutic targets in autoimmune diseases.

## Introduction

B cells are at the core of the adaptive humoral immune system. One of the main features of B cells is to secrete antigen-specific antibody. B cells undergo VDJ gene recombination and somatic hypermutation (SHM) processes to yield a diverse array of B cell receptors (BCRs) enabling recognition of a wide spectrum of antigens (1, 2). The collection of individual BCRs is called the BCR repertoire and reflects the state of the immune system (3, 4). Due to its complexity, it was difficult to obtain information on the exact sequence of the BCR region in a large scale. However, advancements in sequencing technology have facilitated the acquisition of intricate BCR sequence data (5). BCR repertoire analysis has revealed abnormalities in repertoire in patients with autoimmune diseases at the level of B cell subsets, which is useful for understanding the diseases and investigating therapeutic targets. This article summarizes what is currently known about BCR repertoire analysis in autoimmune diseases.

## BCR repertoire analysis

BCR constitutes a tetrameric membrane-bound protein composed of two heavy chains and two light chains, each possessing variable and constant regions. Recombination of the genes encoding the variable region (IGHV, IGHD, and IGHJ in the heavy chains and IGLV and IGLJ in the light chains), deletion or insertion of nucleotides in the junction and subsequent SHM create diversity in the structure of the variable region, allowing the production of antibodies to a variety of foreign antigens (6). Within the variable region, three complementarity-determining regions (CDR) directly engage with antigens. Among these, CDR3 displays the utmost diversity and plays the most important role for antigen specificity (7). A comprehensive characterization of BCRs holds the key to a deeper comprehension of the immune landscape.

Recent advancements in high-throughput sequencing technology have revolutionized the accurate reading of BCR sequences (8). Reading the sequence of BCRs can provide information on the characteristics of BCR repertoire from various perspectives. The CDR3 amino acid sequence can be assumed to be unique to each clone, allowing us to estimate the clonality and diversity of BCR repertoire. Also, chemical or physical features of CDR3 sequences, such as length, hydrophobicity and charge, can represent the repertoire property. Gene usage, meaning the relative fraction of V (or D, J) gene in the repertoire, can also reflect its characteristics. By comparing the nucleotide sequences with germline sequence, we can estimate the degree of SHM (9, 10). Each of these features has been associated with infection, immunization and autoimmunity (11, 12).

## BCR repertoire abnormalities in autoimmune diseases

Most of autoimmune diseases are characterized by the break of immune tolerance to self-antigens and the emergence of autoantibodies. Thus, identification of autoreactive BCR clone and elucidation of the mechanisms which lead to its emergence has been of great interest in this research field. Studies have mainly focused on the specific autoreactive clones and have successfully identified the disease specific features of autoantibodies (13, 14). Recently, some studies have also tried to characterize the global repertoire abnormality in immune-mediated diseases (15, 16).

Systemic lupus erythematosus (SLE) is a systemic disease that affects a variety of organs including skin, kidney, and central nervous system (17). Although the pathogenesis of SLE has not been fully understood, autoreactive B cells and autoantibodies are thought to be important factors in its pathogenesis. There is a bias in the usage of IGHV genes compared to healthy individuals, including increased usage of the IGHV4 gene family (18, 19). Among IGHV4 family, various lines of evidence support the association of IGHV4-34 gene with disease pathogenesis. First, germline sequence of IGHV4-34 has a property of self-antigen binding and usually eliminated from memory B cell subset by negative selection (20). Second, the quantity of IGHV4-34 antibodies with 9G4 idiotype, defined by conserved germline AVY and QW amino acid motifs in the framework-1 region, is elevated in SLE patients (13). Third, in the longitudinal analysis of SLE patient BCR repertoire, usage of IGHV4-34 was increased in the acute state (21). Also, in our recent study of more than 100 SLE patients, IGHV4-34 usage, especially with 9G4 idiotype, showed significant association with disease activity. Interestingly, prominent association was observed in unswitched memory B (USM B), but not in other memory subsets, suggesting the critical role of USM B in its pathogenesis (16) (also see the next section).

Together with gene usage, CDR3 length has long been used as an index of repertoire skewness. In general, CDR3 length is relatively long in naïve B cells, but such long CDR3 has more autoreactivity and thus negatively selected during its maturation to memory B cells (12). In SLE patients, CDR3 length of class-switched memory B cells and plasmablasts were longer than healthy controls, suggesting the breakdown of peripheral checkpoint (15, 16). On the contrary, CDR3 length of naïve B cells were shorter in SLE patients than healthy controls in our recent study (16). As CDR3 length was also short in non-productive CDR3 sequence and strongly correlated with interferon signature in transcriptome, we hypothesized that high interferon activity in bone marrow could affect the early development of B cells (16). As exemplified here, CDR3 length should allow us some biological interpretation of repertoire abnormality. Besides, cell-type dependent features of CDR3 length could explain the controversial reports about the CDR3 length in SLE (22–26) based on the observation in unsorted B cells or peripheral blood mononuclear cells.

The BCR isotype of SLE also exhibited distinctive features. An over representation of IgA and an increased switching to IgE was reported (15). As IgA plays a major role in immune defense at the mucosal surfaces and the frequently used IGHV in SLE has high affinity for microbial antigens (20), the presence of unknown drivers of the disease in the mucosal microbiome has been suggested.

Rheumatoid arthritis (RA) is an autoimmune disease with systemic manifestations typified by chronic arthritis, mainly in the synovial membrane. Based on the therapeutic efficacy of rituximab in patients with RA and the association of IGHV1-69 polymorphisms with disease susceptibility in RA (27, 28), B cells could play a pivotal role for its pathogenesis. Indeed, increased usage of the IGHV4 family has been observed in RA patients (25, 29, 30). In single-cell BCR repertoire analysis of synovial tissues, clonal expansion of age-associated B cells and memory B cells were observed (31). In addition, glycosylation of the variable domain of anti-citrullinated protein antibodies was important for its autoreactivity and activity (32), suggesting the importance of post-translational modification as a feature of BCR repertoire.

Systemic sclerosis (SSc) is an idiopathic autoimmune disease characterized by fibrosis of the skin and various organs. B cells are reported to be involved in the pathogenesis of SSc based on altered B cell subset frequency in disease and the efficacy of B cell-targeted therapy (33, 34). The usage of IGHV genes in SSc patients was different from HC and clonotype was more diverse (35). Particularly in SSc-PAH patients, a decrease in the usage of IGHV2-5 and an increase in SHM frequency in expanded clones were observed (36). CDR3 length in peripheral blood mononuclear cells (PBMC) of SSc patients was significantly shorter (35).

Sjögren’s syndrome (SS) is an autoimmune disease characterized by reduced exocrine function. In SS patients, peripheral B cell abnormalities, including a predominance of naive B cells and a decrease in memory B cells, have been reported (37). Similar to other autoimmune diseases, skewed IGHV gene usage has been reported (38). While CDR3 length remained unchanged (38), there was an increased ratio of non-synonymous mutations in the CDR region of switched memory B cells and USM Bs (36). In naive B cells, an accumulation of self-reactive clones was observed, suggesting impairment in early B cell tolerance checkpoints (36).

ANCA-associated vasculitis (AAV) is an autoimmune disease characterized by inflammation of small- and medium-sized blood vessels. Given the involvement of anti-neutrophil cytoplasmic antibodies (ANCA) in AAV (39), it is expected that there may be some distinct features in the BCR. However, the repertoire of AAV showed no significant differences in the clonal expansion nor diversification (15). Perhaps the limited number of pathogenic ANCA clones in PBMC may explain the lack of detectable differences (15).

Reports of B cell repertoire analysis of major autoimmune diseases are summarized below (**Table 1**).

**Table 1:** BCR repertoire features about several autoimmune diseases.

	SLE	RA	SSc	SS
**IGHV gene**	•Increased usage of IGHV4 gene family (18, 19)	•Association of IGHV1-69 polymorphisms with disease susceptibility (27)	•Biased IGHV gene usage (35) •Reduced IGHV2-5 usage in SSc-PAH (36)	•Altered IGHV gene usage (38)
**CDR3 feature**	•Shorter or longer CDR3 length (22–26)	•Longer or unchanged CDR3 length (25, 29)	•Significantly shortened CDR3 length (35)	•No significant difference in length (38) •Increased non-synonymous mutations in CDR regions of switched and unswitched memory B cells (40)
**Other features**	•Increase in IgA clones and class switch from IgA to IgE (15)	•BCR clonotypes in bone marrow and synovium may be protected from depletion (41)	•Increased SHM frequency in expanded clones in SSc-PAH (36)	•Accumulation of self-reactive clones in naïve B cells (40)

## BCR repertoire abnormalities in CD27^+^IgD^+^ unswitched memory B cell in autoimmune diseases

The characteristics of BCR repertoire vary by B cell subset, including VDJ gene usage, CDR3 length and isotype frequency (42–45). From our BCR repertoire analysis of sorted B cell subsets, fraction of autoreactive clonotypes in USM B showed a prominent correlation with disease activity in SLE (16), illuminating the disease association of this subset. USM Bs represent a category of innate-like B cells responsible for natural IgM production (46). Natural IgM plays a protective role against autoimmunity by removing apoptotic cells and foreign antigens and suppressing innate inflammation (47, 48).

The origin of USM Bs is still a matter of debate. Some have argued for its correspondence with marginal zone B cells (49, 50), while others have reported the GC-independent origin early in the primary response (51). Still, some argued that they are GC-dependent B cells (50, 52). USM Bs could be further divided into functionally distinctive populations based on IgM positivity (53) or T-bet positivity (54). It remains unclear whether different classes of USM Bs have different origins or if each has multiple origins.

Populational and functional abnormalities of USM B in patients with autoimmune diseases have been reported (55–57). It has been hypothesized that USM B becomes exhausted in the inflammatory milieu, leading to decreased IgM production (55). This is in line with the clinical observation that patients with SLE have low levels of IgM, and that the decreased number of IgM correlates with the duration of disease (58). In RA, USM B was reported to have a proinflammatory feature, decreased capacity of IgM production and altered VDJ gene usage (55). As well, the total number of USM B was decreased but recovered after the effective therapy (55). SSc also showed fewer memory B cell subsets, particularly in USM B, in the peripheral blood (57). In SS, there was a significant decrease in memory B cells, including USM B, in the peripheral blood, while there was a local accumulation of these cells in the salivary glands. This accumulation may possibly be associated with activation or enhancement of local autoimmune responses (56). These reports support the role of USM B in autoimmune diseases.

In recent article (16), we reported that usage of many VDJ genes was skewed in USM B of SLE and other autoimmune diseases compared to healthy controls. We noticed that those differentially used VDJ genes were largely correspondent with the differentially used genes between USM B and naïve B cells in healthy controls. In principal component analysis based on VDJ gene usage, gene usage pattern of USM B in SLE and other diseases were significantly skewed toward the direction into naïve B cells. Gene usage in plasmablasts of SLE was skewed into the same direction as well in the same coordinate plane. For further understanding, we developed a composite score of VDJ gene usage to quantify this skewing. Interestingly, a strong correlation between the skew level of plasmablast gene usage and that of USM B gene usage across patients was observed. These observations implied a shared pathology between these two subsets of SLE.

Also, this gene usage-based score showed significant correlation with the SHM frequency in plasmablast and peripheral helper T cell transcriptome signature, which is associated with GC-independent maturation of B cells (59), in T helper 1 cells. Thus, we proposed that the activation of extrafollicular pathway in SLE could result in the global skew of gene usage of these two subsets.

**Figure 1:**
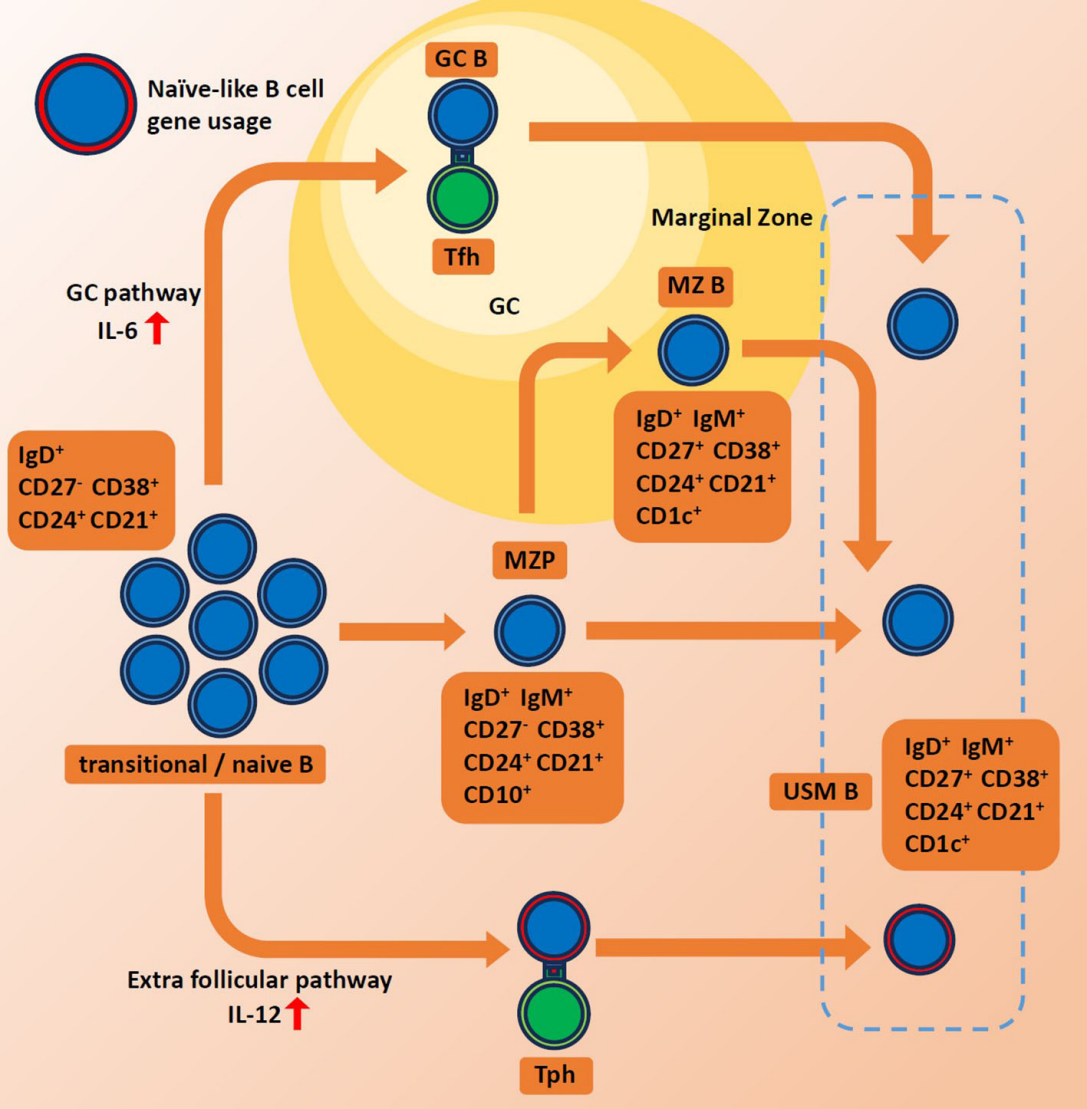
Schematics for maturation of unswitched memory B cells. Red arrows indicate high levels of each interferon. GC, germinal center. MZ B, marginal zone B cell. MZP, marginal zone precursor B cell. Tph, peripheral helper T cell. Tfh, follicular helper T cell. USMB, unswitched memory B cell.

While much of the research interest has been focused on the extrafollicular maturation of plasmablasts, we have noted that the extrafollicular maturation of USM Bs could also potentially play interesting roles in autoimmune diseases. At steady-state conditions, the IGHV4-34 gene is preferentially expressed in naive B cells. However, it tends to be eliminated in memory B cells through GC. Extrafollicular (GC-independent) pathway is reported to be activated in SLE, bacterial infection as well as COVID-19 infections (21, 60–64). The extrafollicular pathway often bypasses the germinal center maturation process and is known to produce rapid and short-lived antibody responses. The rapid and modestly regulated nature of the extrafollicular pathway could allow for the preferential survival and expansion of IGHV4-34-expressing memory B cells (**Figure 1**). Additionally, we observed increased *TBX21* expression among presumably extrafollicular pathway driven USM B, suggesting its distinctive function. However, the association between elevated IGHV4-34 usage and other features of USM B in autoimmune diseases, such as decreased population size and decreased IgM production, remains uncertain.

Despite the presence of many unknowns, including population heterogeneity, USM Bs have the potential to hold the key to understanding autoimmune diseases.

## BCR repertoire as a treatment target and biomarker

B-cell-targeted therapy has already been widely employed in the treatment of autoimmune diseases. Understanding the target of clinically used treatments can guide us toward developing new treatment strategies. For instance, belimumab, a human monoclonal antibody that inhibits B cell stimulating factors, is one of only two biologics approved by the United States Food and Drug Administration for the treatment of SLE. Belimumab treatment has been observed to reduce the fraction of IGHV4-34 in unmutated IgM sequences (65). In line with it, we observed that patients with SLE after treatment with belimumab showed decreased degree of extrafollicular pathway in USM B, which resulted in decreased use of IGHV4-34 (16).

Rituximab (RTX) is the mainstay of B-cell depletion therapy and is widely used to treat autoimmune diseases such as RA, AAV, and idiopathic inflammatory muscle disease (IIM) (66–70). In RA patients, dominant clones before treatment are rapidly cleared from the peripheral blood after treatment, while persistence of clones predicts subsequent non-response (41). In AAV and SLE patients, the majority of clones persistent after RTX treatment was class-switched (15). On the contrary, mycophenolate mofetil (MMF), which belongs to antimetabolites that inhibit purine synthesis in cells, has reduced class-switched clones and increased USM Bs, suggesting the different targets of these drugs on BCR repertoire (15).

Intravenous immunoglobulin (IVIg) acts against activated immune cells and signaling pathways, resulting in anti-inflammatory and immune-modifying effects (71). In patients with IIM, pre-treatment dominant clones disappear with IVIg treatment and the higher the cumulative frequency of pre-treatment dominant clones, the better the response to treatment. It is suggested that treatment responsiveness to IVIg may depend on the composition of the BCR repertoire prior to treatment (72).

Together, different drugs target different aspects of BCR repertoire. Consequently, the BCR repertoire can serve as an indicator of treatment efficacy and a predictor of treatment responsiveness.

Diagnosis of diseases by repertoire analysis is also expected. As a promising example, patients with IgA Nephropathy (IgAN) and healthy individuals could be distinguished based on the repertoire status (73). IgAN is a type of glomerulonephritis, an immune-related disease characterized by renal deposition of IgA (74). BCR repertoire analysis of the B cells of IgAN patients showed shortened CDR3 length. In addition, disease-associated IGH clones were identified, allowing classification of IgAN and healthy individuals. IgAN is generally diagnosed by renal biopsy, which is an invasive test. Less invasive diagnosis and monitoring of disease status may be possible through repertoire analysis.

Another report has successfully classified patients with celiac disease according to the results of repertoire analysis (75).

## Conclusions and perspectives

In this article, we have conducted a review of BCR repertoire analysis of autoimmune diseases, with a specific focus on USM Bs. Within the BCR repertoire of autoimmune diseases, there is a noticeable bias in the usage of VDJ genes. This bias likely reflects underlying B cell biology, such as the activation of extrafollicular pathways. The study of B cell repertoires in autoimmune diseases is still in its early stages. Large-scale repertoire analysis, including single-cell analysis and the incorporation of clinical information, holds the potential to provide further insights and understanding in this field.

USM Bs represent an under-studied population in the context of autoimmunity. Nevertheless, several observations provide support for their potential role in autoimmune diseases. While the precise role of USM Bs remains incompletely understood, it is plausible that a subset of USM Bs, which have matured via the extrafollicular pathway, may contribute to pathogenic processes. Additionally, quantitative and qualitative defects in USM Bs may impair the functionality of protective IgM, potentially leading to disease development.

The results of BCR repertoire analysis have provided various insights into the pathogenesis of autoimmune diseases. However, the BCR repertoire analyses that have been performed so far are mainly conducted on bulk samples. Recently, single-cell repertoire analysis, which can examine BCR information on a single cell basis, has been attracting attention (76). Single-cell repertoire analysis allows pairing of heavy and light chains. For example, a technique for high-throughput mapping of BCR sequences by pairing heavy and light chains has been developed (77, 78). While detailed analysis is undoubtedly crucial, the importance of conducting large-scale cohort studies should not be underestimated, given the heterogeneous nature of autoimmune diseases. Future efforts aimed at a comprehensive analysis of the BCR repertoire in a large cohort will unquestionably enhance our understanding of these conditions.

## Author contributions

HY: Writing – original draft. TI: Writing – review & editing. YN: Writing – review & editing. MO: Writing – review & editing. KF: Supervision, Writing – review & editing.

## References

[1] Alt FW Yancopoulos GD Blackwell TK Wood C Thomas E Boss M. Ordered rearrangement of immunoglobulin heavy chain variable region segments. EMBO J (1984) 3(6):1209–19. doi: 10.1002/j.1460-2075.1984.tb01955.x PMC557501 6086308

[2] Papavasiliou FN Schatz DG. Somatic hypermutation of immunoglobulin genes: merging mechanisms for genetic diversity. Cell (2002) 109 Suppl:S35–44. doi: 10.1016/s0092-8674(02)00706-7 11983151

[3] Liu X Wu J. History, applications, and challenges of immune repertoire research. Cell Biol Toxicol (2018) 34(6):441–57. doi: 10.1007/s10565-018-9426-0 29484527

[4] Shay T Kang J. Immunological Genome Project and systems immunology. Trends Immunol (2013) 34(12):602–9. doi: 10.1016/j.it.2013.03.004 PMC4615706 23631936

[5] Boyd SD Joshi SA. High-throughput DNA sequencing analysis of antibody repertoires. Microbiol Spectr (2014) 2(5). doi: 10.1128/microbiolspec.AID-0017-2014 26104353

[6] McHeyzer-Williams M Okitsu S Wang N McHeyzer-Williams L. Molecular programming of B cell memory. Nat Rev Immunol (2011) 12:24–34. doi: 10.1038/nri3128 22158414 PMC3947622

[7] Miles JJ Douek DC Price DA. Bias in the αβ T-cell repertoire: implications for disease pathogenesis and vaccination. Immunol Cell Biol (2011) 89(3):375–87. doi: 10.1038/icb.2010.139 21301479

[8] Campbell PJ Pleasance ED Stephens PJ Dicks E Rance R Goodhead I. Subclonal phylogenetic structures in cancer revealed by ultra-deep sequencing. Proc Natl Acad Sci USA (2008) 105(35):13081–6. doi: 10.1073/pnas.0801523105 PMC2529122 18723673

[9] Vander Heiden JA Yaari G Uduman M Stern JN O’Connor KC Hafler DA. pRESTO: a toolkit for processing high-throughput sequencing raw reads of lymphocyte receptor repertoires. Bioinformatics (2014) 30(13):1930–2. doi: 10.1093/bioinformatics/btu138 PMC4071206 24618469

[10] Gupta NT Vander Heiden JA Uduman M Gadala-Maria D Yaari G Kleinstein SH. Change-O: A toolkit for analyzing large-scale B cell immunoglobulin repertoire sequencing data. Bioinformatics (2015) 31(20):3356–8. doi: 10.1093/bioinformatics/btv359 PMC4793929 26069265

[11] Zheng B Yang Y Chen L Wu M Zhou S. B-cell receptor repertoire sequencing: Deeper digging into the mechanisms and clinical aspects of immune-mediated diseases. iScience (2022) 25(10):105002. doi: 10.1016/j.isci.2022.105002 36157582 PMC9494237

[12] Nielsen SCA Boyd SD. Human adaptive immune receptor repertoire analysis-Past, present, and future. Immunol Rev (2018) 284(1):9–23. doi: 10.1111/imr.12667 29944765

[13] Richardson C Chida AS Adlowitz D Silver L Fox E Jenks SA. Molecular basis of 9G4 B cell autoreactivity in human systemic lupus erythematosus. J Immunol (2013) 191(10):4926–39. doi: 10.4049/jimmunol.1202263 PMC3816606 24108696

[14] Sokolove J Johnson DS Lahey LJ Wagner CA Cheng D Thiele GM. Rheumatoid factor as a potentiator of anti-citrullinated protein antibody-mediated inflammation in rheumatoid arthritis. Arthritis Rheumatol (2014) 66(4):813–21. doi: 10.1002/art.38307 PMC3994896 24757134

[15] Bashford-Rogers RJM Bergamaschi L McKinney EF Pombal DC Mescia F Lee JC. Analysis of the B cell receptor repertoire in six immune-mediated diseases. Nature (2019) 574(7776):122–6. doi: 10.1038/s41586-019-1595-3 PMC6795535 31554970

[16] Ota M Nakano M Nagafuchi Y Kobayashi S Hatano H Yoshida R. Multimodal repertoire analysis unveils B cell biology in immune-mediated diseases. Ann Rheum Dis (2023) 82(11):1455–63. doi: 10.1136/ard-2023-224421 37468219

[17] Arbuckle MR McClain MT Rubertone MV Scofield RH Dennis GJ James JA. Development of autoantibodies before the clinical onset of systemic lupus erythematosus. N Engl J Med (2003) 349(16):1526–33. doi: 10.1056/NEJMoa021933 14561795

[18] Silberstein LE Jefferies LC Goldman J Friedman D Moore JS Nowell PC. Variable region gene analysis of pathologic human autoantibodies to the related i and I red blood cell antigens. Blood (1991) 78(9):2372–86. doi: 10.1182/blood.V78.9.2372.2372 1657249

[19] Pascual V Victor K Lelsz D Spellerberg MB Hamblin TJ Thompson KM. Nucleotide sequence analysis of the V regions of two IgM cold agglutinins. Evidence that the VH4-21 gene segment is responsible for the major cross-reactive idiotype. J Immunol (1991) 146(12):4385–91. 1710250

[20] Schickel JN Glauzy S Ng YS Chamberlain N Massad C Isnardi I. Self-reactive VH4-34-expressing IgG B cells recognize commensal bacteria. J Exp Med (2017) 214(7):1991–2003. doi: 10.1084/jem.20160201 28500047 PMC5502416

[21] Tipton CM Fucile CF Darce J Chida A Ichikawa T Gregoretti I. Diversity, cellular origin and autoreactivity of antibody-secreting cell population expansions in acute systemic lupus erythematosus. Nat Immunol (2015) 16:755–65. doi: 10.1038/ni.3175 PMC4512288 26006014

[22] Liu S Hou XL Sui WG Lu QJ Hu YL Dai Y. Direct measurement of B-cell receptor repertoire's composition and variation in systemic lupus erythematosus. Genes Immun (2017) 18(1):22–7. doi: 10.1038/gene.2016.45 28053320

[23] Yurasov S Wardemann H Hammersen J Tsuiji M Meffre E Pascual V. Defective B cell tolerance checkpoints in systemic lupus erythematosus. J Exp Med (2005) 201(5):703–11. doi: 10.1084/jem.20042251 PMC2212839 15738055

[24] Meffre E Milili M Blanco-Betancourt C Antunes H Nussenzweig MC Schiff C. Immunoglobulin heavy chain expression shapes the B cell receptor repertoire in human B cell development. J Clin Invest (2001) 108(6):879–86. doi: 10.1172/JCI13051 PMC200933 11560957

[25] Zhang Y Lee TY. Revealing the immune heterogeneity between systemic lupus erythematosus and rheumatoid arthritis based on multi-omics data analysis. Int J Mol Sci (2022) 23(9):5166. doi: 10.3390/ijms23095166 35563556 PMC9101622

[26] Robins H. Immunosequencing: applications of immune repertoire deep sequencing. Curr Opin Immunol (2013) 25:646–52. doi: 10.1016/j.coi.2013.09.017 24140071

[27] Vencovský J Zd'árský E Moyes SP Hajeer A Ruzicková S Cimburek Z. Polymorphism in the immunoglobulin VH gene V1-69 affects susceptibility to rheumatoid arthritis in subjects lacking the HLA-DRB1 shared epitope. Rheumatol (Oxford) (2002) 41(4):401–10. doi: 10.1093/rheumatology/41.4.401 11961170

[28] Edwards JC Szczepanski L Szechinski J Filipowicz-Sosnowska A Emery P Close DR. Efficacy of B-cell-targeted therapy with rituximab in patients with rheumatoid arthritis. N Engl J Med (2004) 350(25):2572–81. doi: 10.1056/NEJMoa032534 15201414

[29] Doorenspleet ME Klarenbeek PL de Hair MJ van Schaik BD Esveldt RE van Kampen AH. Rheumatoid arthritis synovial tissue harbours dominant B-cell and plasma-cell clones associated with autoreactivity. Ann Rheum Dis (2014) 73(4):756–62. doi: 10.1136/annrheumdis-2012-202861 23606709

[30] Voswinkel J Pfreundschuh M Gause A. Evidence for a selected humoral immune response encoded by VH4 family genes in the synovial membrane of a patient with RA. Ann N Y Acad Sci (1997) 815:312–5. doi: 10.1111/j.1749-6632.1997.tb52072.x 9186667

[31] Dunlap G Wagner A Meednu N Zhang F Jonsson A Wei K. Clonal associations of lymphocyte subsets and functional states revealed by single cell antigen receptor profiling of T and B cells in rheumatoid arthritis synovium. bioRxiv (2023) 03:18.533282. doi: 10.1101/2023.03.18.533282

[32] Kissel T Ge C Hafkenscheid L Kwekkeboom JC Slot LM Cavallari M. Surface Ig variable domain glycosylation affects autoantigen binding and acts as threshold for human autoreactive B cell activation. Sci Adv (2022) 8(6):eabm1759. doi: 10.1126/sciadv.abm1759 35138894 PMC8827743

[33] Mavropoulos A Simopoulou T Varna A Liaskos C Katsiari CG Bogdanos DP. Breg cells are numerically decreased and functionally impaired in patients with systemic sclerosis. Arthritis Rheumatol (2016) 68:494–504. doi: 10.1002/art.39437 26414243

[34] Giuggioli D Lumetti F Colaci M Fallahi P Antonelli A Ferri C. Rituximab in the treatment of patients with systemic sclerosis. Our experience and review of the literature. Autoimmun Rev (2015) 14:1072–8. doi: 10.1016/j.autrev.2015.07.008 26209905

[35] Shi X Shao T Huo F Zheng C Li W Jiang Z. An analysis of abnormalities in the B cell receptor repertoire in patients with systemic sclerosis using high-throughput sequencing. PeerJ (2020) 8:e8370. doi: 10.7717/peerj.8370 31988805 PMC6968515

[36] de Bourcy CFA Dekker CL Davis MM Nicolls MR Quake SR. Dynamics of the human antibody repertoire after B cell depletion in systemic sclerosis. Sci Immunol (2017) 2(15):eaan8289. doi: 10.1126/sciimmunol.aan8289 28963118 PMC5800854

[37] Hansen A Daridon C Dörner T. What do we know about memory B cells in primary Sjögren's syndrome? Autoimmun Rev (2010) 9(9):600–3. doi: 10.1016/j.autrev.2010.05.005 20452465

[38] Hou X Hong X Ou M Meng S Wang T Liao S. Analysis of gene expression and TCR/B cell receptor profiling of immune cells in primary sjögren's syndrome by single-cell sequencing. J Immunol (2022) 209(2):238–49. doi: 10.4049/jimmunol.2100803 35705251

[39] Geetha D Jefferson JA. ANCA-associated vasculitis: core curriculum 2020. Am J Kidney Dis (2020) 75(1):124–37. doi: 10.1053/j.ajkd.2019.04.031 31358311

[40] Corsiero E Sutcliffe N Pitzalis C Bombardieri M. Accumulation of self-reactive naïve and memory B cell reveals sequential defects in B cell tolerance checkpoints in Sjögren's syndrome. PloS One (2014) 9(12):e114575. doi: 10.1371/journal.pone.0114575 25535746 PMC4275206

[41] Pollastro S Klarenbeek PL Doorenspleet ME van Schaik BDC Esveldt REE Thurlings RM. Non-response to rituximab therapy in rheumatoid arthritis is associated with incomplete disruption of the B cell receptor repertoire. Ann Rheum Dis (2019) 78(10):1339–45. doi: 10.1136/annrheumdis-2018-214898 PMC6788876 31217169

[42] Mroczek ES Ippolito GC Rogosch T Hoi KH Hwangpo TA Brand MG. Differences in the composition of the human antibody repertoire by B cell Subsets in the blood. Front Immunol (2014) 5:96. doi: 10.3389/fimmu.2014.00096 24678310 PMC3958703

[43] Kaplinsky J Li A Sun A Coffre M Koralov SB Arnaout R. Antibody repertoire deep sequencing reveals antigen-independent selection in maturing B cells. Proc Natl Acad Sci USA (2014) 111:E2622–9. doi: 10.1073/pnas.1403278111 PMC4078805 24927543

[44] Samuels J Ng YS Coupillaud C Paget D Meffre E. Impaired early B cell tolerance in patients with rheumatoid arthritis. J Exp Med (2005) 201(10):1659–67. doi: 10.1084/jem.20042321 PMC2212916 15897279

[45] Mietzner B Tsuiji M Scheid J Velinzon K Tiller T Abraham K. Autoreactive IgG memory antibodies in patients with systemic lupus erythematosus arise from nonreactive and polyreactive precursors. Proc Natl Acad Sci USA (2008) 105(28):9727–32. doi: 10.1073/pnas.0803644105 PMC2474524 18621685

[46] Baumgarth N Herman OC Jager GC Brown L Herzenberg LA Herzenberg LA. Innate and acquired humoral immunities to influenza virus are mediated by distinct arms of the immune system. Proc Natl Acad Sci USA (1999) 96:2250–5. doi: 10.1073/pnas.96.5.2250 PMC26769 10051627

[47] Zhou ZH Tzioufas AG Notkins AL. Properties and function of polyreactive antibodies and polyreactive antigen-binding B cells. J Autoimmun (2007) 29:219–28. doi: 10.1016/j.jaut.2007.07.015 PMC2100422 17888628

[48] Quartier P Potter PK Ehrenstein MR Walport MJ Botto M. Predominant role of IgM-dependent activation of the classical pathway in the clearance of dying cells by murine bone marrow-derived macrophages *in vitro* . Eur J Immunol (2005) 35:252–60. doi: 10.1002/eji.200425497 15597324

[49] Weller S Braun MC Tan BK Rosenwald A Cordier C Conley ME. Human blood IgM “memory” B cells are circulating splenic marginal zone B cells harboring a prediversified immunoglobulin repertoire. Blood (2004) 104:3647–54. doi: 10.1182/blood-2004-01-0346 PMC2590648 15191950

[50] Sanz I Wei C Jenks SA Cashman KS Tipton C Woodruff MC. Challenges and opportunities for consistent classification of human B cell and plasma cell populations. Front Immunol (2019) 10:2458. doi: 10.3389/fimmu.2019.02458 31681331 PMC6813733

[51] Taylor JJ Pape KA Jenkins MK. A germinal center-independent pathway generates unswitched memory B cells early in the primary response. J Exp Med (2012) 209(3):597–606. doi: 10.1084/jem.20111696 22370719 PMC3302224

[52] Seifert M Przekopowitz M Taudien S Lollies A Ronge V Drees B. Functional capacities of human IgM memory B cells in early inflammatory responses and secondary germinal center reactions. Proc Natl Acad Sci USA (2015) 112(6):E546–55. doi: 10.1073/pnas.1416276112 PMC4330750 25624468

[53] Koelsch K Zheng NY Zhang Q Duty A Helms C Mathias MD. Mature B cells class switched to IgD are autoreactive in healthy individuals. J Clin Invest (2007) 117(6):1558–65. doi: 10.1172/JCI27628 PMC1866247 17510706

[54] Johnson JL Rosenthal RL Knox JJ Myles A Naradikian MS Madej J. The transcription factor T-bet resolves memory B cell subsets with distinct tissue distributions and antibody specificities in mice and humans. Immunity (2020) 52(5):842–855.e6. doi: 10.1016/j.immuni.2020.03.020 32353250 PMC7242168

[55] Hu F Zhang W Shi L Liu X Jia Y Xu L. Impaired CD27^+^IgD^+^ B cells with altered gene signature in rheumatoid arthritis. Front Immunol (2018) 9:626. doi: 10.3389/fimmu.2018.00626 29628928 PMC5877504

[56] Hansen A Odendahl M Reiter K Jacobi AM Feist E Scholze J. Diminished peripheral blood memory B cells and accumulation of memory B cells in the salivary glands of patients with Sjögren's syndrome. Arthritis Rheumatol (2002) 46(8):2160–71. doi: 10.1002/art.10445 12209521

[57] Simon D Balogh P Bognár A Kellermayer Z Engelmann P Németh P. Reduced non-switched memory B cell subsets cause imbalance in B cell repertoire in systemic sclerosis. Clin Exp Rheumatol (2016) 34 Suppl 100(5):30–6. 27056741

[58] Sivri A Hascelik Z. IgM deficiency in systemic lupus erythematosus patients. Arthritis Rheum (1995) 38:1713. doi: 10.1002/art.1780381128 7488297

[59] Rubtsova K Rubtsov AV Thurman JM Mennona JM Kappler JW Marrack P. B cells expressing the transcription factor T-bet drive lupus-like Autoimmunity. J Clin Invest (2017) 127:1392–404. doi: 10.1172/JCI91250 PMC5373868 28240602

[60] Elsner RA Shlomchik MJ. Germinal center and Extrafollicular B cell responses in vaccination, immunity, and Autoimmunity. Immunity (2020) 53:1136–50. doi: 10.1016/j.immuni.2020.11.006 PMC7748291 33326765

[61] William J Euler C Christensen S Shlomchik MJ. Evolution of autoantibody responses via somatic hypermutation outside of germinal centers. Science (2002) 297:2066–70. doi: 10.1126/science.1073924 12242446

[62] Rao DA Gurish MF Marshall JL Slowikowski K Fonseka CY Liu Y. Pathologically expanded peripheral T helper cell subset drives B cells in rheumatoid arthritis. Nature (2017) 542:110–4. doi: 10.1038/nature20810 PMC5349321 28150777

[63] Woodruff MC Ramonell RP Nguyen DC Cashman KS Saini AS Haddad NS. Extrafollicular B cell responses correlate with neutralizing antibodies and morbidity in COVID-19. Nat Immunol (2020) 21(12):1506–16. doi: 10.1038/s41590-020-00814-z PMC7739702 33028979

[64] Jenks SA Cashman KS Zumaquero E Marigorta UM Patel AV Wang X. Distinct effector B cells induced by unregulated toll-like receptor 7 contribute to pathogenic responses in systemic lupus erythematosus. Immunity (2018) 49(4):725–739.e6. doi: 10.1016/j.immuni.2018.08.015 30314758 PMC6217820

[65] Huang W Quach TD Dascalu C Liu Z Leung T Byrne-Steele M. Belimumab promotes negative selection of activated autoreactive B cells in systemic lupus erythematosus patients. JCI Insight (2018) 3(17):e122525. doi: 10.1172/jci.insight.122525 30185675 PMC6171800

[66] Aggarwal R Oddis CV Goudeau D Koontz D Qi Z Reed AM. Autoantibody levels in myositis patients correlate with clinical response during B cell depletion with rituximab. Rheumatol (Oxford) (2016) 55(6):991–9. doi: 10.1093/rheumatology/kev444 PMC6281030 26888854

[67] Salliot C Finckh A Katchamart W Lu Y Sun Y Bombardier C. Indirect comparisons of the efficacy of biological antirheumatic agents in rheumatoid arthritis in patients with an inadequate response to conventional disease-modifying antirheumatic drugs or to an anti-tumour necrosis factor agent: a meta-analysis. Ann Rheum Dis (2011) 70:266–71. doi: 10.1136/ard.2010.132134 21097801

[68] Stone JH Merkel PA Spiera R Seo P Langford CA Hoffman GS. Rituximab versus cyclophosphamide for ANCA-associated vasculitis. N Engl J Med (2010) 363(3):221–32. doi: 10.1056/NEJMoa0909905 PMC3137658 20647199

[69] Jones RB Tervaert JW Hauser T Luqmani R Morgan MD Peh CA. Rituximab versus cyclophosphamide in ANCA-associated renal vasculitis. N Engl J Med (2010) 363(3):211–20. doi: 10.1056/NEJMoa0909169 20647198

[70] Dass S Rawstron AC Vital EM Henshaw K McGonagle D Emery P. Highly sensitive B cell analysis predicts response to rituximab therapy in rheumatoid arthritis. Arthritis Rheum (2008) 58:2993–9. doi: 10.1002/art.23902 18821683

[71] Miyasaka N Hara M Koike T Saito E Yamada M Tanaka Y. Effects of intravenous immunoglobulin therapy in Japanese patients with polymyositis and dermatomyositis resistant to corticosteroids: a randomized double-blind placebo-controlled trial. Mod Rheumatol (2012) 22(3):382–93. doi: 10.1007/s10165-011-0534-4 PMC3375426 21971943

[72] Anang DC Walter HAW Lim J Niewold I van der Weele L Aronica E. B-cell receptor profiling before and after IVIG monotherapy in newly diagnosed idiopathic inflammatory myopathies. Rheumatol (Oxford) (2023) 62(7):2585–93. doi: 10.1093/rheumatology/keac602 PMC10321087 36321862

[73] Huang C Li X Wu J Zhang W Sun S Lin L. The landscape and diagnostic potential of T and B cell repertoire in Immunoglobulin A Nephropathy. J Autoimmun (2019) 97:100–7. doi: 10.1016/j.jaut.2018.10.018 30385082

[74] Wyatt RJ Julian BA. IgA nephropathy. N Engl J Med (2013) 368(25):2402–14. doi: 10.1056/NEJMra1206793 23782179

[75] Shemesh O Polak P Lundin KEA Sollid LM Yaari G. Machine learning analysis of naïve B-cell receptor repertoires stratifies celiac disease patients and controls. Front Immunol (2021) 12:627813. doi: 10.3389/fimmu.2021.627813 33790900 PMC8006302

[76] Haque A Engel J Teichmann SA Lönnberg T. A practical guide to single-cell RNA-sequencing for biomedical research and clinical applications. Genome Med (2017) 9(1):75.525. doi: 10.1186/s13073-017-0467-4 28821273 PMC5561556

[77] Setliff I Shiakolas AR Pilewski KA Murji AA Mapengo RE Janowska K. High-throughput mapping of B cell receptor sequences to antigen specificity. Cell (2019) 179(7):1636–1646. e1615. doi: 10.1016/j.cell.2019.11.003 31787378 PMC7158953

[78] Walker LM Shiakolas AR Venkat R Liu ZA Wall S Raju N. High-throughput B cell epitope determination by next-generation sequencing. Front Immunol (2022) 13:855772. doi: 10.3389/fimmu.2022.855772 35401559 PMC8984479

